# Re-Visiting of Plentiful Food Sources and Food Search Strategies in Desert Ants

**DOI:** 10.3389/fnins.2012.00102

**Published:** 2012-07-05

**Authors:** Harald Wolf, Matthias Wittlinger, Siegfried Bolek

**Affiliations:** ^1^Institute of Neurobiology, University of UlmUlm, Germany

**Keywords:** desert ant *Cataglyphis*, navigation, feeding site assessment, path integration, error compensation

## Abstract

North African desert ants, *Cataglyphis fortis*, are established model organisms in animal navigation research. *Cataglyphis* re-visit plentiful feeding sites, but their decision to return to a feeder and the organization of food searches has been little studied. Here we provide a review of recent advances regarding this topic. At least two parameters determine the ants’ assessment of site quality, namely, amount of food available and reliability of food encounter on subsequent visits. The amount of food appears to be judged by the concentration of items at the food uptake site. Initially the amount of food in a feeder dominates the foragers’ decision to return, whereas learning about reliability takes precedence in the course of a few visits. The location of a worthwhile site is determined by the animals’ path integration system. In particular, the distance of the feeding site is memorized as the arithmetic average of the distances covered during the previous outbound and homebound journeys. Feeding sites that are small and inconspicuous cannot be approached directly with sufficient certainty, due to inevitable inaccuracies of the path integrator. Instead, desert ants steer downwind of the goal to encounter the odor plume emanating from the food and they follow this plume to the feeder. The angle steered downwind reflects the animals’ maximal navigation error and is adjusted according to experience. In summary, food searches of desert ants provide an unexpected wealth of features that may advance our understanding of search, navigation, and decision strategies. There are several aspects that warrant further scrutiny.

## Introduction

The life of animals, including more “simple” invertebrates, abounds with decisions, most of which have a bearing on reproductive fitness or even survival. And while the individual decision may not be too important, a balanced strategy for arriving at viable decisions in the long term is certainly essential. Food acquisition is a good example here since it has direct consequences for survival and reproduction. When a forager encounters a plentiful feeding site it cannot fully exploit, is it useful to return later? Or are there better chances of finding food elsewhere, due to high food abundance or because other foragers will have removed the bounty next time around?

Desert ants are good study objects in this context because, firstly, food availability is easily manipulated in the barren and open desert habitat and, secondly, appreciation of food sources by the ants can be measured quantitatively as the focusing of their food search behavior. Moreover, the North African species *Cataglyphis fortis* is a well-studied model system in navigation research (Wehner, 2003), with good associated knowledge of behavioral aspects. For instance, the ants may be trained to re-visit plentiful feeders, a property regularly employed in navigation research. Feeder location is determined by a path integrator that keeps track of a forager’s position with respect to its nest throughout foraging excursions (Wehner and Wehner, 1986; Müller and Wehner, 1988; Wehner and Srinivasan, 2003).

By contrast, comparatively little is known about either the parameters used by the ants in evaluating whether a food source is valuable enough to re-visit or about other features associated with food searches. We thus provide an overview of recent results with a focus on the following questions.

1. What prompts the ants to re-visit a feeding site in the first place? Is it the amount of food or the reliability of food encounter on sequential visits?
2. Is it the previous outbound or the last inbound journey that is used to establish the memory of the feeding site location?
3. In the case of small and inconspicuous food sources, is the accuracy of the path integrator sufficient to find the food source again? And if not, what strategies are used for a reliable encounter?

We use these recent data to identify important points for further study in this area.

## What Prompts Desert Ants to Return to a Feeding Site?

### Food amount and reliability of food encounter

We examined whether it is the amount of food available on the previous visit or the reliability of food encounters on sequential visits that influences the return to a feeding site by *Cataglyphis* ants (Bolek et al., 2012b). Only novice foragers were used in these experiments to avoid any influence from previous experience.

#### Experimental situations

**Figure 1:**
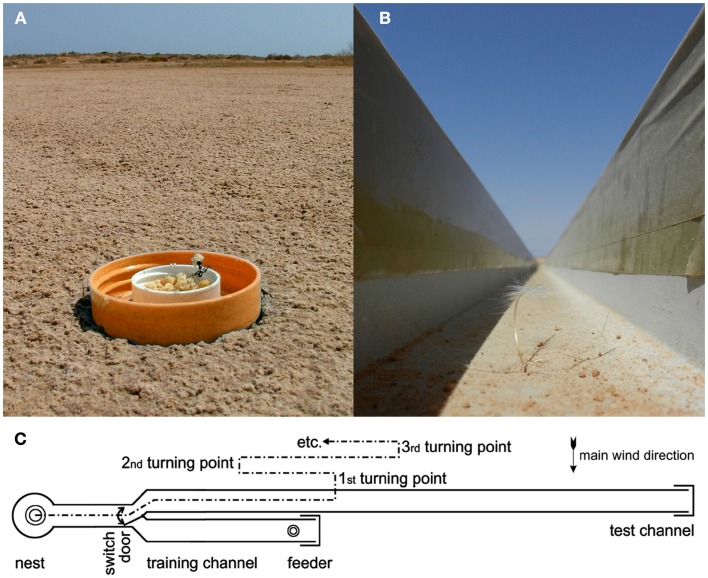
**Experimental feeding station (A) and channel arrangement (B,C)**. **(A)** A feeding station on the desert floor is visited by a *Cataglyphis fortis* forager (Forel 1902; Wehner, 1983). The lid of a marmalade jar is usually pressed into the desert floor (rather than left lying on top as shown here for clarity) to avoid any visual cues extending above desert floor level. The lid captures any food crumbs that are blown out of the small central food container or that are removed by the foragers but dropped during sampling of different items. **(B)** View along a training channel (width 7 cm, walls 7 cm high) from the nest entrance. Tape covers on the walls provide a slippery surface that dissuades most ants from escaping from the channel, thus increasing the number of animals that find the feeder. **(C)** Arrangement of training (bottom) and test (top) channels and their connection to the nest via a Y-junction with a switch door.

**Figure 2:**
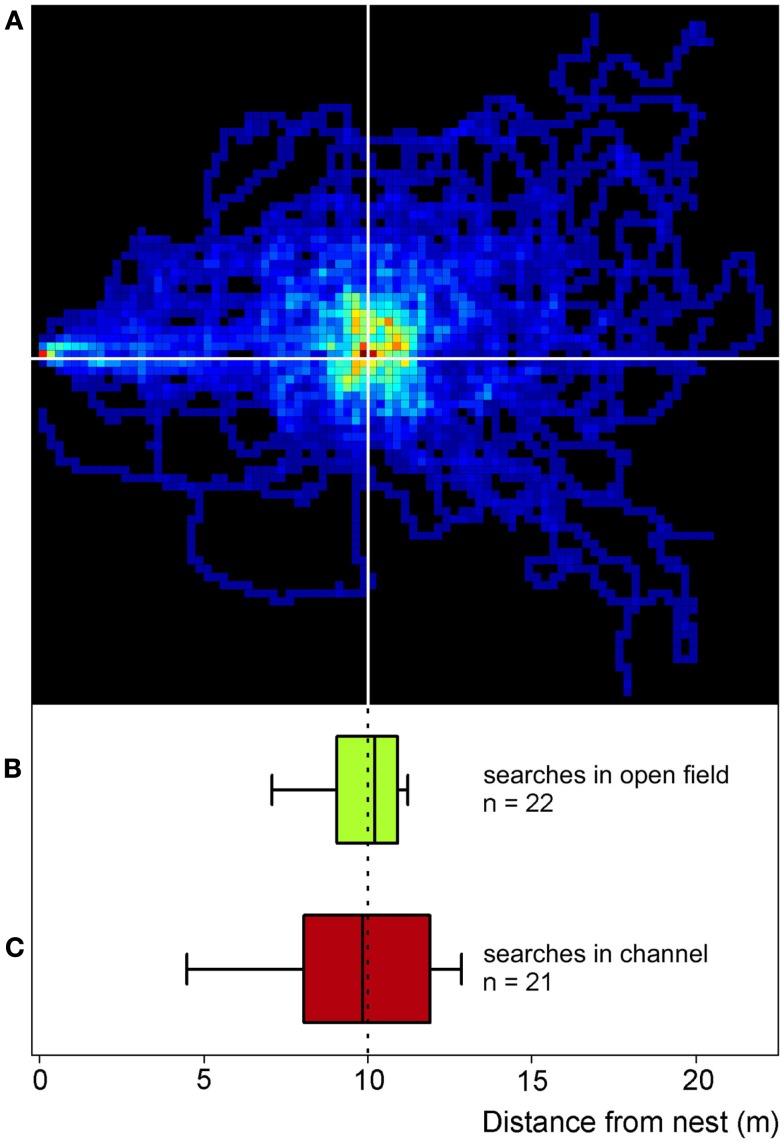
**Desert ants’ search behavior in the open field (compare Figure 1A) and in channels (compare Figures 1B,C)**. For the two-dimensional search density plot in **(A)**, the number of ants’ visits to each 25 cm × 25 cm pixel of the feeder surrounds was recorded, summed, and normalized to the maximum number of visits per pixel in the plot. The darkest red represents the highest density (100%), the darkest blue just a single visit, and black areas were not visited at all (0%). Recordings lasted for 2.5 min after an animal had left the nest (note red pixel on the left hand margin); nest-feeder distance was 10 m. The ants (*n* = 31) had visited the full feeder (>800 biscuit crumbs) once before the recordings were made. To construct the box plot in **(B)**, the data in **(A)** were projected onto the nest-feeder axis, i.e., any movements along the axis perpendicular to the nest-feeder direction were disregarded. Like in the channel experiments [see **(C)**], the initial six turning points on the nest – feeder axis were used to calculate medians and percentiles (below; *n* = 22, since not all 31 ants performed six turns in the projected path as required for the analysis). The box plot in **(C)** presents searches recorded in the test channel used in all the other experiments described in this report (Figure 1C). The ants had visited a full feeder once in the training channel (as in the open field) before the recordings were made. Note the similarity of the plots in **(B,C)**, attesting to comparable search behavior in the channel and in the open field. Box plots show medians, box margins (+75th, −25th percentiles) and whiskers (+90th, −10th percentiles) in this and all following figures.

**Figure 3:**
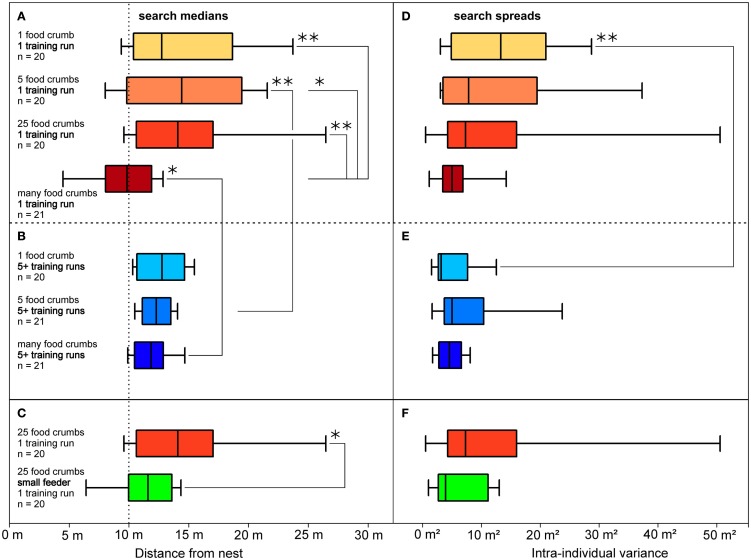
**Distributions of food searches (A,D), according to their dependency on the amount of food presented, and (B,E) according to rewarded experience with the feeding site**. **(A,D)** Data from ants that had performed a single (training) visit to a feeder located in a channel, 10 m from the nest. The experimental groups differed in the amount of food available in the feeder, as noted on the left and indicated by the box color (darker colors represent more food items). Also noted are numbers of experimental animal. Boxes and whiskers as in Figure 2. Significant differences are indicated by brackets and asterisks; one asterisk, *p* < 0.05; two asterisks, *p* < 0.01; absence of significant difference is not indicated. **(B,E)** Data from ants that had performed five or more (training) visits; the experimental groups differed in the amount of food available in the feeder; other labels as in **(A)**. **(C,F)** Data from ants that had visited a feeder equipped with 25 food items once before being tested. The feeder was either of standard size (32 mm diameter; same situation as in **(A)**, bright red box) or small (8 mm, green box); food density was thus 16-fold higher in the small feeder. Other labels as in **(A)**. Search medians are plotted on the left **(A–C)**, search spreads on the right **(D–F)** as variances of the individuals’ searches.

Experiments in artificial channels make the recording of quantitative data much easier compared to the open desert terrain. It was thus first necessary to establish whether or not food searches performed in channels do indeed reflect normal search behavior as performed in the open field (Figure 2). Food searches were therefore initially recorded in the open desert terrain by placing a feeder 10 m from the nest (Figure 1A) and recording the ants’ foraging trips by means of a 2-m by 2-m grid painted on the desert floor around the feeder. When an ant had encountered a full feeder on its first trip to the feeding site, its next food search was clearly centered on the previous position of the now absent feeder (Figure 2A). This demonstrates that, in this situation at least, the ants memorize the vector to the food site quite exactly. When projecting the two-dimensional search trajectory onto the nest-feeder axis (details in legend Figure 2, see also Bolek et al., 2012a), the resulting search distribution (Figure 2B) was similar to the search pattern recorded in a channel under otherwise identical conditions (Figure 2C; same data set as Figure 3A, bottom box). This observation attests to the validity of the channel experiments carried out in the following experiments.

**Figure 4:**
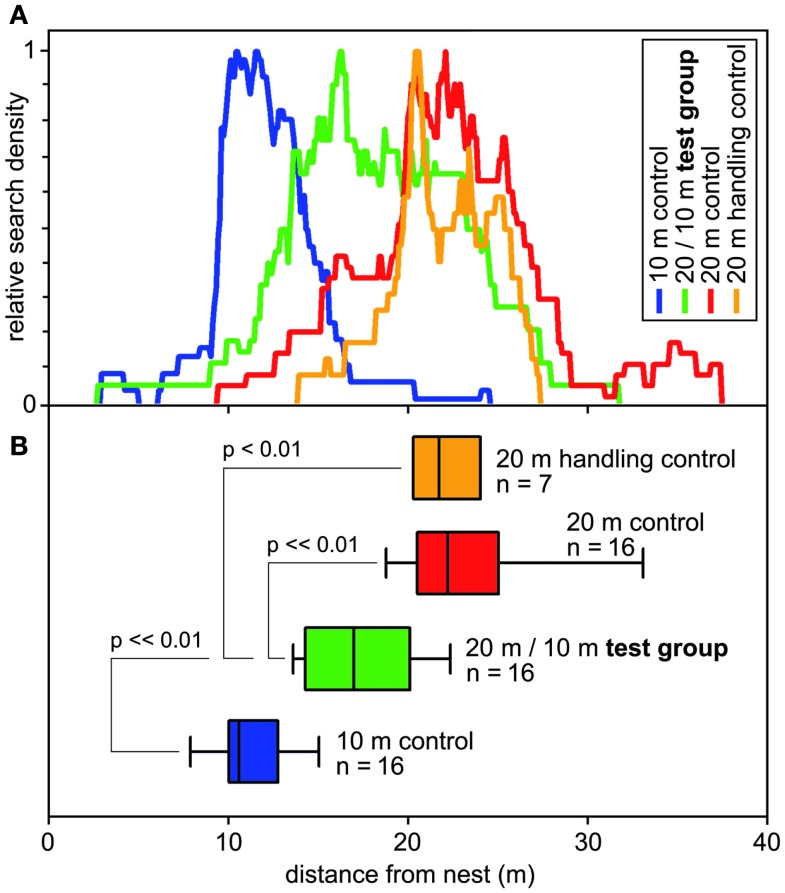
**Search behavior of desert ants on their outbound journey, from the nest to the site of a feeder (that was removed for testing)**. **(A)** Normalized search densities (no. of visits per 10 cm bin of test channel). Color code noted in top right inset. **(B)** Corresponding box-and-whisker plots (boxes and whiskers as in Figure 2); abscissa, distance from the nest. Color code corresponds to **(A)**; indicated are numbers of animals and significant differences including significance levels. Medians of the turning points of the food searches of the ant individuals were used to calculate ANOVAs, with pair-wise comparisons according to Holm–Sidak *post hoc* test.

The setup for the channel experiments in this and the subsequent experiments (including those in section Is it the Previous Outbound or the Last Inbound Journey that Establishes the Memory of the Feeding Site Location?) consisted of two parallel channels that were both connected to the nest via a Y-shaped junction (Figures 1B,C). In the training channel, a feeder was established at 10 m distance from the ants’ nest. The channel arrangement increased the number of ants foraging at the feeder by restricting their foraging excursions to the channel, and it also facilitated the recording of search behavior. For testing, the ants were led into the test channel that extended for more than 20 m beyond the feeder in parallel to the training channel. A switch door in the channel near the nest allowed selection of the ants to be tested (Figure 1C). The ants’ search behavior was recorded by noting their U-turns in the test channel. For each ant individual, search medians (Figures 2B,C, 3, and 4B) and spreads (Figures 3D–F) were calculated from the initial six turns. Spreads were calculated as variances of the individuals’ searches. For the individuals’ values, means, and percentiles were determined for the experimental groups.

To test what prompts an ant to search for a food site, the ants were left to find the feeder by chance (similar to the situation in Figure 1A, though in the training channel). In the different test situations, the feeder was equipped with either one, five, 25, or many (>800) food morsels. Once an ant had visited the feeder and returned to the nest with a food morsel, the next foraging trip was recorded in the test channel (Figures 3A,D). This experimental series thus examined the effect of different food amounts in the feeder on search behavior. Alternatively, a minimum number of five visits were allowed before recording the search (Figures 3B,E). This experimental series examined the effect of the foragers’ experience with the food site on search behavior. The recordings of all ants in a given experimental situation were used to calculate search medians (box-and-whisker plots, Figures 2B,C and 3) and spreads (Figures 3D–F).

#### Experimental results

The experiments demonstrated that both parameters, the amount of food in the feeder, and experience regarding reliability of food encounter, influence the desert ants’ search for the feeding site. Ants that had encountered the feeder just once (Figures 3A,D) exhibited rather different search patterns upon their next visit, depending on the amount of food presented in the feeder. Different from the initial chance encounter, this second visit appeared goal-oriented, as indicated by the more or less narrow search distributions in Figure 3D. Searches for a feeder with just a few food items or only a single food item had search centers noticeably beyond the original feeder position and larger spreads (Figures 3A,D, top three boxes). This is not at all surprising, since the respective ant had removed much or all food from the feeder on its previous visit and should not necessarily expect further morsels in that particular location, reflecting the typical situation for *C. fortis* foragers that usually scavenge on scattered insect carcasses (Wehner et al., 1983). These searches appear to reflect sector fidelity as reported previously (Wehner et al., 1983; Schmid-Hempel, 1984), with the search extending roughly into the previously successful direction but without a clear search for the previous food location. When the feeder was equipped with many (>800) standardized biscuit crumbs (ca. 1.5 by 1.5 mm in size; Figures 3A,D, bottom box), the searches were much more focused and the search center coincided almost exactly with the previous feeder location.

A larger number of successful visits had similar effects on search density as had food abundance, i.e., repeated successful visits overrode the effect of abundance just described. If the ants were allowed to visit the feeder 5 or more times before being tested, all searches were well-focused just beyond the previous feeder position (Figures 3B,E), even if the feeder had yielded just a single item on each previous visit (Figures 3B,E, top box; quantitative data on search densities in Bolek et al., 2012b).

In summary, the ants assess both food abundance and the reliability of food encounter. Increases in both parameters lead to more focused searches for the food source, with learning about reliability overriding food abundance after several visits. It is an important additional result that desert ants *C. fortis* exhibit a well-defined food vector (Figure 2; see also below, Figure 4), in addition to the sector fidelity reported previously (Wehner et al., 1983; Schmid-Hempel, 1984). Sector fidelity appears to be applied for single food items that are removed upon being met by the forager. If food is left back at the food site or food is encountered reliably over several visits, the ants show well-focused searches for a familiar food source, thus memorizing a food vector. The observation of such point fidelity in a quantitative manner is an important novel finding in *Cataglyphis*. Although point fidelity is routinely employed when training ants to visit a feeding site, this aspect had as yet received almost no attention, in contrast to the well-known sector fidelity (Wehner et al., 1983; Schmid-Hempel, 1984; see also Buchkremer and Reinhold, 2008). In summary, point fidelity appears to be used for feeders that are worthwhile re-visiting due to large food supply or high reliability, while sector fidelity would appear to represent the normal mode of foraging for isolated prey items such as scattered arthropod carcasses.

The emergence of a food vector after sufficient reinforcement further demonstrates that experience shapes the ants’ food search behavior. This is interesting when one considers that the same navigational toolkit is employed as when determining the home vector, but the home vector does not improve or otherwise change with increasing experience (Merkle et al., 2006).

### Assessment of food sources, in *Cataglyphis* and other species

A preliminary experiment indicates that the ants may judge the food amount not by counting items – which would not be expected anyway (Franks et al., 2006) – but by assessing the density of food items at the location of food uptake (Bolek et al., 2012b). Figures 3C,F illustrate that *Cataglyphis’* food search becomes more focused if 25 food items are offered in a small feeder of 8 mm diameter, rather than in the standard feeder of 32 mm diameter that is used in all other experiments. This increases the density of food items 16-fold, which is the only change that should be noticeable to the ants under the experimental conditions (Bolek et al., 2012b).

It has yet to be established how the animals assess density. Mechanosensory input from legs and mouthparts is an obvious possibility, as is food odor, because a higher concentration of odorants would be expected to emanate from a higher density of food items, even at some distance. It is further conceivable that the visit to a plentiful feeder initiates associative learning in desert ants, similar to the situation in honeybees (Pelz et al., 1997). This is a viable option since associative learning in response to odor stimuli has recently been reported in *Camponotus* ants (Guerrieri and d’Ettorre, 2010) and appears also to be present in *Cataglyphis* according to preliminary experiments (Klein, 2011; Wohlfarth, 2011). Pelz et al. (1997) have shown that odorant concentration has an influence on associative learning in honeybees. When extrapolating the findings in honeybees to *Cataglyphis*, food odorants represent a conditioning stimulus that is associated with the food reward. The conditioned response may be the food vector that takes the ant back to the previously visited food source in this case. And differences in odor concentration may make a plentiful food source a more intense and more salient olfactory stimulus than a poor food source. By the same line of argument, repeated successful visits to the food source may represent repeated conditioning trials in an associative learning process, sharpening the conditioned response, i.e., focusing the food search. Such an interpretation sees decisions in the light of reward-dependent learning, a topic considered in detail in other contributions to this issue.

The Australian desert ant, *Melophorus bagoti*, occupies an ecological niche very similar to that of the Saharan *Cataglyphis*. Comparison of two species that have evolved their desert life independently is thus tempting, although there are few studies as yet on food site vectors in either species, *Melophorus* or *Cataglyphis*. A recent study by Schultheiss and Cheng (in review) demonstrated that *Melophorus* adjust their search behavior differently for protein and carbohydrate foods, with carbohydrate food eliciting more concentrated searches (compare data on *Formica schaufussi* (Traniello et al., 1992; Fourcassié and Traniello, 1993, below). This corresponds to the natural distribution of these food supplies, with carbohydrates offered mainly in plentiful patches by fruiting plants and proteins occurring primarily as scattered arthropod carcasses. Apart from this adaptive search layout, *Melophorus*’ food search patterns are centered on the familiar food site, resembling the well-known searches for the nest.

Assessment of feeding sites in *Cataglyphis* appears comparable to the food assessment strategies in other ants. A major difference concerns the fact that most ant species use their social organization to exploit food sources through recruitment of nest mates. Such group foraging allows adjustment of deployed forager forces to feeding site yield, for instance (e.g., in *Monomorium pharaonis*; Sumpter and Beekman, 2003). Recruitment is absent in the desert ant, *C. fortis*, which only forages individually.

The amount of food available is judged in ants by parameters such as satiation (e.g., in *Lasius niger*; Mailleux et al., 2000) or portability of larger items (e.g., in *Pheidole pallidula*; Detrain and Deneubourg, 1997). If a large amount of food is encountered, nest mates are usually recruited by laying pheromone trails on the return journey to the nest. Other recruiting mechanisms are also observed, however, including the leading of novice foragers to promising feeding sites in tandem runs (e.g., in *Temnothorax albipennis*; Franks and Richardson, 2006). Indeed, recruitment is often used to measure the assessment of feeding sites by ant species in experimental paradigms. And while pheromone trails eliminate the need for establishing food vectors, the tandem-running *Temnothorax*, and scouts of other species will need to memorize the location of a food supply for future return visits. As is the case with *Cataglyphis*, this aspect has, as yet, received little attention.

Other species, such as *F. schaufussi*, do not exhibit noticeable assessment of food amount (Robson and Traniello, 1998) but appear to focus primarily on food quality (Traniello et al., 1992; Fourcassié and Traniello, 1993), for example, regarding protein, carbohydrate, and fat contents (see also Schultheiss and Cheng, in review for *Melophorus*, above). Learning of the reliability of a food supply or assessment of food amount was not observed in this species, and the evaluation of food quality also requires further scrutiny. What the ants consider high-quality food may vary, depending on the time of the year, the food naturally available at a given time, requirements of the brood, and other factors, even though no such variations were observed in *Formica schafussi* (Traniello et al., 1992). These complications may as yet have prevented a detailed study of this aspect.

## Is it the Previous Outbound or the Last Inbound Journey that Establishes the Memory of the Feeding Site Location?

*Cataglyphis* desert ants primarily use path integration to navigate in their desert habitat while foraging (Wehner and Srinivasan, 2003), although landmarks (e.g., Wehner et al., 1996), ground structures (Seidl and Wehner, 2006), and odor marks (Steck et al., 2009, 2010) are also used if available. The ants use a skylight compass (Wehner, 1997; Wehner and Müller, 2006) and a stride integrator (Wittlinger et al., 2006, 2007) to monitor their meandering search paths and constantly update distance and direction back to the nest. The ants also use their path integrator to return to plentiful or reliable feeding sites (Wolf and Wehner, 2000). It has remained unclear, however, what is used to memorize the feeder position: is it the straight homebound path from the feeder or the state of the path integrator when finding the food, i.e., on the outbound journey, or are these two measures combined, and if so, in what manner?

These questions were addressed (Bolek et al., 2012a) by connecting an ants’ nest to a U-shaped metal channel, as described above (Figures 1B,C). A plentiful feeder was placed in this training channel at 20 m (or 10 m, below) distance from the nest. A much longer test channel was arranged just next to and in parallel to the training channel. This setup allowed selective assessment of the distance component, or odometer, of the ants’ path integrator. Once an ant had visited the feeder and returned to the nest with a food morsel, its next foraging trip was monitored by leading it into the (empty) test channel *via* a switch door. These (control) ants searched for food quite reliably close to the previous nest-feeder distance of 20 m (Figure 4). This was to be expected, not least on the basis of the preceding experiments on feeder assessment (Figure 3A, bottom box; see also Figure 2). In the following set of experiments, the distance of the experimental animals’ homebound journey was altered by gently catching them at the feeder once they had taken up a food item, and releasing the ants closer to the nest, at half the outbound distance, i.e., 10 m. One would expect these animals to concentrate their searches for food at around 10 m if they took their homebound journey for memorizing the feeder position, or at around 20 m if they took their outbound journey.

The data in Figure 4 demonstrate that the ants follow neither of these expectations but rather average the out- and inbound path lengths. Furthermore, they consider the linear average, instead of the harmonic average or other averaging options, at least in the present experimental situation. It will be interesting to see if, with more typical outbound search trajectories, i.e., that are meandering and much longer than the straight inbound path, the weighting of the two legs of the foraging trip changes.

The present result corresponds well to the few previous reports on food site vectors, particularly Cheng and Wehner (2002). Similar observations were made in honeybees (Otto, 1959), although more recent contradictory results also exist for bees (Srinivasan et al., 1997). In the latter report, estimation of the distance to a feeder was examined by a forager honeybee’s return journey to that feeder under controlled artificial laboratory conditions (rather than observing the bee’s dance back in the hive). Experimentally interfering with the bees’ optic flow odometer demonstrated that outbound travel distance apparently determines the bee’s memory of nest-feeder distance. Similar to the situation in desert ants, however, more natural foraging situations may possibly reveal additional mechanisms. This may be particularly true when considering that odometer information appears to be determined in different ways for a forager bee’s own return travel to a feeder and for its dance communication to fellow foragers back in the hive (Dacke and Srinivasan, 2008).

## Is the Accuracy of the Path Integrator Sufficient to Find the Food Source Again?

Any navigation system has a limited accuracy, although present technical systems may be very precise. In the case of desert ant navigation, the accuracy is surprisingly good, considering the meandering foraging paths, large foraging distances, and inherent errors in the path integrator as a dead reckoning system (Müller and Wehner, 1988). This accuracy is not sufficient, however, to steer precisely toward a goal smaller than a few degrees in azimuth without landmarks or other structures supporting orientation (Wolf and Wehner, 2000, 2005; Wolf, 2008). This is acceptable for *Cataglyphis* ants for two reasons. Firstly, the immediate nest surroundings will be familiar to a forager both after its initial few trips and also from refuse deposition and short exploratory outings by the ant before assuming its foraging task (Wehner et al., 2004). This ensures finding of the nest on return journeys even in the nest location is not met spot-on. Secondly, the ants possess a number of backup strategies, including olfactory orientation (Wolf and Wehner, 2000) and an efficient search strategy (Wehner and Srinivasan, 1981; Merkle and Wehner, 2010). Nonetheless, navigation inaccuracy may present a problem for returning to a plentiful feeding site, at least initially.

### Downwind approach strategy

**Figure 5:**
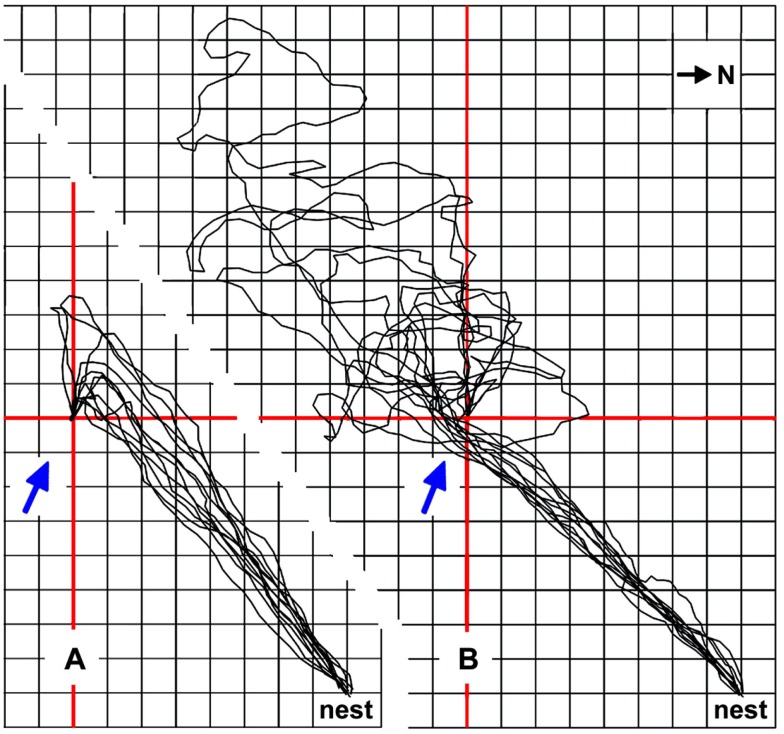
**The downwind approach strategy exhibited by *Cataglyphis* ants reduces the average foraging path length**. Typical downwind approaches of four foragers are shown in **(A)**, the final upwind segments guided by the odor traces emanating from the small and inconspicuous feeder (see Figure 1A). Feeder position is marked by red cross lines; grid line distance is 0.5 m. Ambient wind direction is indicated by blue arrows. The downwind approach afforded an additional 1.6 m walking distance compared to a beeline approach, on average. **(B)** The same ants occasionally choose a slightly different path, probably due to lack of experience and slight shifts in wind direction (Wolf, 2008). Approaches of the same four animals, performed just before or after the sample runs shown in **(A)** and leading into an area upwind of the food source, are superimposed in **(B)**. They demonstrate that the ants missed the feeder by passing upwind of the odor traces and illustrate the ensuing searches of an average 9.7 m walking distance, in addition to the direct approach distance of about 5.7 m. Compass north is indicated in the top right corner; nest-feeder distance is 5.7 m.

*Cataglyphis* ants minimize these problems by using other cues to localize a food source, if such cues happen to be available. Landmarks are an obvious possibility (e.g., Wehner et al., 1996), and odors are another important cue (Steck et al., 2009, 2010). Desert ant food – typically arthropod carcasses, or occasionally biscuit crumbs from experimenting biologists – will normally exude some odor. This odor not only alerts the ants to novel food items over a distance (Wehner and Duelli, 1971), it also allows guided approaches to a familiar feeding site. The ants usually steer downwind of a known food source to encounter the odor plume emanating from the food (similar to the situation depicted in Figure 5A). Once the ants have encountered the plume, it will safely guide them to the food source. Such a strategy affords a detour and thus a longer path than a direct approach. In return, it avoids missing the goal or small food locations in particular. With a direct approach, the ants would inadvertently walk into an area upwind of the food in about 50% of cases due to navigation inaccuracies. The resultant searches are usually much longer than the detour required by the downwind strategy (Figure 5B; Wolf, 2008). In the sample traces shown in Figure 5B, the average detour required by the downwind approach extends the walking path by about 28% of the nest-feeder distance, i.e., from 5.7 m nest-feeder distance to 7.3 m walking trajectory. The search trajectory initiated after passing the food on the upwind side is an average of twice (198%) the nest-feeder distance, extending the approach from 5.7 m nest-feeder distance to an average 17.0 m walking trajectory. Despite some effort, the downwind approach strategy thus appears clearly advantageous.

### Error compensation

**Figure 6:**
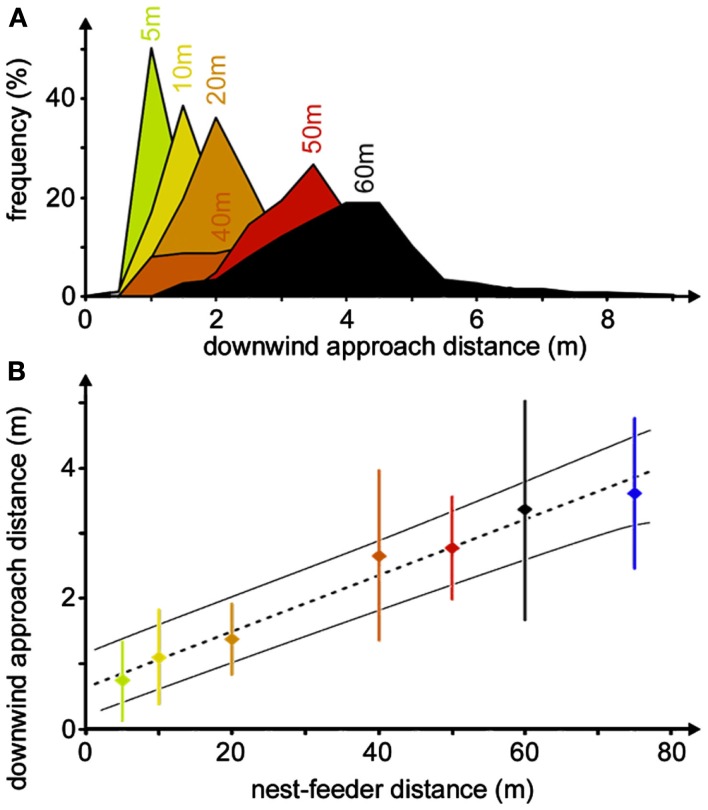
**Compensation of navigation inaccuracy by downwind approach**. **(A)** Distributions of downwind approach distances for different nest-feeder distances. Histograms were recorded at the nest-feeder distances noted above the peak bins (bin widths 0.5 m). Different histograms are distinguished by different colors. For the different histograms, the numbers of ant individuals were between 8 and 29, yielding between 42 and 747 recordings, except for 75 m nest-feeder distance with only three ants and six recordings (see Wolf and Wehner, 2005). **(B)** The same data set is shown as a plot of downwind approach distance against nest-feeder distance. Dotted line indicates the best-fit regression, thin lines mark 95% confidence intervals. Measurements for each individual were pooled before calculating means, SD, and regression line. Color code as in **(A)**.

The downwind approach strategy is used by desert ants quite reliably, although adjustment of the downwind distance occurs during the initial four to six visits to a food source (Wolf, 2008). It represents a so-called error compensation strategy (Biegler, 2000), i.e., inevitable navigation errors are compensated for, or rather accounted for, by tailoring the approach strategy to minimize search effort. In the case of the desert ants’ downwind approach, this means that the animals consider their angular steering error by keeping downwind of the assumed goal direction by their expected maximum error angle, which will safely lead them into the downwind area and allow encountering of the food odor. It also means that the distance steered downwind of a food source should increase linearly with nest-feeder distance. And this is indeed borne out when establishing feeding stations at different distances from an ants’ nest, ranging from 5 to 60 m (Figure 6A), or even 75 m (Figure 6B), although ants are difficult to train to such distant feeding sites. The angle steered downwind of the food site is in the range of 4°–8°, which should correspond to the maximum navigation error according to the error compensation strategy (Wolf and Wehner, 2005).

In other words, *Cataglyphis* desert ants are able to judge their own navigation accuracy. Although this knowledge appears to be inexact initially and is adjusted during the first three to five visits to a familiar site (Wolf, 2008), this result is remarkable for an insect navigator. It is particularly unexpected in view of the fact that the typical prey items of *Cataglyphis* are scattered arthropod carcasses that do not warrant a return to the previously visited site.

## Conclusion and Outlook

Food searches in desert ants, *C. fortis*, provide an unexpected wealth of features that may advance our understanding of search, navigation, and learning and decision strategies. More detailed studies would appear promising, particularly for the following aspects: (i) The assessment of food site quality, beyond food abundance and reliability of food encounter; this concerns the chemical quality, the density, or the size of food items and possible learning mechanisms; (ii) the mode of memorizing food site vectors in more typical food searches, with meandering outbound search paths and straight homebound paths; (iii) the downwind approach strategy of desert ants with regard to the adjustment of the downwind distance under different circumstances, such as wind conditions, desert floor structure, presence of landmarks.

## Conflict of Interest Statement

The authors declare that the research was conducted in the absence of any commercial or financial relationships that could be construed as a potential conflict of interest.
